# Cure of Congenital Deafness

**Published:** 1840

**Authors:** 


CURE OF A DEAF MUTE, BY PUNCTURING THE MEMBRANA TYMPANI.

Dr. Prosser, of Jacksonville, Illinois, has sent us an authentic newspaper account of the sudden and permanent acquisition of the sense of hearing, by a congenital deaf mute, Mr. J. Washington, of Winchester in that State, from a puncture by his own hand, of the membrana tympani of one of his ears. Mr. W. is an educated man, and understood, perfectly, what he was about to do. He states, that in making the puncture, with a lancet pointed instrument, concealed in a canula, he had to pass through *two* membranes, one of which he regards as exterior to the head of the drum, and altogether abnormal. If this be a fact, it is probable that his deafness did not depend upon obstruction in the eustachian tubes, and that the puncture of the exterior would of itself have been sufficient. The following is his own account of what followed on the operation:

"The pain of the operation combined with the sudden admission of air, like a peal of thunder, immediately threw me into a series of spasms, and for that night my fate seemed suspended like a pendulum, 'twixt life and death; but by the attention of Doctor Wilson and others, I have so far recovered, as to be able to prosecute the study of elocution, under N. M. Knapp, Esq.

One or two little things struck me at the time as peculiar; one of which is, that all inanimate things contained within themselves latent sounds, which might be produced by striking them—another is, that before the operation I could remember the exact expression of my own face, so as to compare it with others, and draw comparisons between them, but since then it is impossible for me to recollect my own countenance. I account for it from the fact that mutes have always a statue-like, stolid, asinine, imperturbable look, but now there is an ever varying, undefinable meaning, that cannot be retained in the mind; another is loss of powers of application, and although the memory of events or things that happened before is as good as ever, yet it is extremely difficult to remember what has passed since the operation." D.

